# Content and timing of the 6–8 week maternal postnatal check: a mixed-methods study

**DOI:** 10.3399/BJGPO.2024.0229

**Published:** 2025-04-09

**Authors:** Clare Macdonald, Fiona Cross-Sudworth, Laura Quinn, Christine MacArthur, Debra Bick, Ellie Jones, Beck Taylor

**Affiliations:** 1 Department of Applied Health Sciences, University of Birmingham, Birmingham, UK; 2 Warwick Medical School, University of Warwick, Coventry, UK; 3 Warwick Clinical Trials Unit, Warwick Medical School, University of Warwick, Coventry, UK

**Keywords:** women’s health, obstetrics, maternal health, mental health, general practitioners, primary healthcare

## Abstract

**Background:**

Since 2020, the General Medical Services contract requires GP practices in England to offer women a GP appointment 6–8 weeks after birth: the ‘6–8 week postnatal check’ or ‘consultation’. Historically, provision of checks was variable, and women still frequently report poor experiences.

**Aim:**

To explore GPs’ and women’s perspectives of the 6–8 week postnatal check, including key components and timing.

**Design & setting:**

A mixed-methods study was undertaken with focus groups of GPs and women, and an online survey of GPs in England.

**Method:**

Focus groups explored GPs’ and women’s experiences of postnatal consultations. An online survey explored GPs’ clinical approach, organisation, and improvement potential. Quantitative analysis examined associations between demographics and clinical approach. Thematic framework analysis was used for qualitative data.

**Results:**

In total, 18 women and 14 GPs participated in focus groups; 671 GPs completed the survey. Mental wellbeing and contraception were reported as important topics, although some women were not asked about mental health. GP survey responses indicated most recommendations from national guidance were ‘always’ or ‘very often’ covered by most, but not all GPs. Clinical coverage was higher for GPs who used clinical templates, had awareness of guidance, were female, or were a parent. Many GPs (*n* = 326/670, 49%) needed more time than they were allocated for the consultation (*n* = 524/670 [78%] allocated ≤15 minutes; *n* = 351/670 [52%] completed in ≤15 minutes).

**Conclusion:**

This study suggests GPs are allocated insufficient time for postnatal consultations, with substantial variation in practice. Specifying consultation duration and consideration of template usage in policy may improve care and outcomes for women.

## How this fits in

Since 2020, GP practices in England have been contractually required and funded to offer all women a GP consultation 6–8 weeks after giving birth; there is national guidance about how to conduct the consultation and what it can involve, but surveys of women report variable, often poor experiences. This mixed-methods study investigated GPs’ and women’s views and experiences of 6–8 week postnatal consultations. GPs and women reported the same clinical areas as being important, particularly mental health and contraception. Neither the guidance, nor the contract, stipulate the duration of the postnatal check; women sometimes felt rushed, while GPs frequently reported needing more time than they were allocated.

### Note on language

Where we use the terms ‘woman’ or ‘women’ in the context of their perinatal care, this is inclusive of all pregnant people or those who have given birth even if they do not identify as women.

## Introduction

All women in England should be offered a routine consultation with a GP about their health 6–8 weeks after giving birth; this was introduced to the GP contract in 2020, with funding provided through an uplift to the global sum payment.^1^ The contract outlines the following four broad areas the consultation should cover: mental health; physical health; contraception; and pelvic health. Detailed guidance on ‘What Good Looks Like’ was published by NHS England (NHSE) in 2023.^2^ Similarly, guidance from National Institute for Health and Care Excellence (NICE) in 2021^3^ recommends women have a GP review 6–8 weeks postnatally and makes detailed recommendations about postnatal clinical requirements for all healthcare professionals, including GPs. Most women have typically been seen by a GP in the weeks and months after birth, including before the recent contractual requirement.^4^ National maternity surveys in 2023 reported variable, sometimes poor, experiences of postnatal care, with Healthwatch reporting 16% not receiving a GP postnatal check and 30% of those who did not being asked about their mental health.^5,6^

Pregnancy, birth, and the early postnatal period can precipitate a range of new physical and mental illnesses. Additionally, more women are living with long-term conditions, including diabetes and obesity,^7^ making their perinatal journey more medically complex. Postnatal mental illness — predominantly depression and anxiety — is common, affecting 10–20% of women.^8,9^ Most maternal deaths in the UK occur postnatally, with mental illness-related causes being common.^10^ During this period, most women are not under specialist or secondary care services; the GP is therefore essential to identify, manage, and refer health problems. GPs value their role in providing postnatal care but face organisational barriers impacting the quality of their care.^11^ Evidence from the 1990s suggested female GPs were more likely to enquire about psychological issues in postnatal checks.^12^ Although research has investigated women’s recollection of the content of postnatal checks,^13^ and our systematic review from 2023 described GPs’ views and experiences of postnatal care,^11^ to our knowledge, no research has investigated GP views or approaches to the postnatal consultation since the introduction of the contractual requirement to provide one in England.

This study aimed to explore GPs’ and women’s perspectives of the postnatal check, including its key components, and timing (currently 6–8 weeks postpartum). Specifically, the aim of the qualitative focus groups was to explore GPs’ and women’s perspectives of the postnatal check and generate survey questions. The aim of the survey was to understand clinical and organisational practices in the provision of postnatal checks in primary care settings in England and identify differences between GP demographic groups. The study also explored facilitators and barriers to GPs providing a consultation in line with NICE guidance^3^ and how GP behaviours, actions, and language impact on women’s experiences. Here, we report on findings relating to the content and timing of the maternal postnatal consultation.

## Method

This was a mixed-methods study in England; the design was selected to provide breadth and depth in understanding this potentially complex consultation. Initial qualitative data analysis from focus groups with women and GPs informed GP survey questions; survey questions were developed based on issues raised in focus groups along with NICE recommendations^3^ (as the standard for clinical approach).

### Focus groups

Focus groups took place May–September 2022. Convenience sampling through Twitter (now known as X) and WhatsApp, via professional networks and snowballing, was used to recruit GPs and GP Specialty Trainees (GPSTs) to four online focus group. Participants received a coffee-shop voucher. A convenience sampling approach recruited women with recent postnatal experience to four in-person focus groups in Birmingham and Leicester (one at an infant-feeding support group, three at a ‘Baby Sensory’ class). Participants received a shopping voucher.

Topic guides for GPs and women and a prompt sheet for women (Supplementary Box 1-2) were developed with public contributors and maternity researchers’ input, informed by NICE guidance.^3^ Focus groups were facilitated by two researchers (CM, FCS).

Recordings were transcribed verbatim, anonymised, checked for accuracy, and imported into NVivo (version 12) software. A thematic framework analysis approach was taken.^14^ Following transcription and familiarisation, three researchers (CM, FCS, BT) conducted initial open coding. They developed a working analytical framework that was applied to the rest of the data, with two researchers (CM and FCS) independently dual coding two transcripts. Data were summarised and charted into an analytical framework matrix. Charted data were reviewed, and analytical summaries prepared to develop themes. The research team met regularly to develop the analysis and interpretation. A predominantly inductive approach was used, drawing on NICE guidance^3^ and a previous qualitative systematic review^11^ to support interpretation.

### Survey

GPs and GPSTs in England were eligible. Recruitment was open; a digital flyer was circulated in multiple ways: West Midlands Clinical Research Network (CRN) disseminated to research active practices and to CRNs in other regions; the survey was shared via social media (Twitter [now known as X], medic Facebook groups, GP WhatsApp groups), via professional networks and snowballing. This strategy was adopted with the aim of gathering the largest and most diverse sample given the demographic heterogeneity of the GP population and GP practices, consistent with other studies in UK primary care.^15^ Survey participants could opt-in to a £50 voucher prize draw. The questionnaire (Supplementary Box 3), comprising 30 questions was hosted at www.onlinesurveys.ac.uk from February–June 2023. Using a combination of Likert scale, multiple choice, and free-text questions, the following four areas were explored: participant demographics; clinical approach; organisation; and potential for improvement.

Free-text responses from the survey were analysed in NVivo (version 12) using the same thematic framework analysis approach as focus groups; the coding framework from focus groups was used, while also exploring survey data for any additional concepts. Data were extracted into an analytic matrix and reviewed alongside the themes developed from the focus groups. Thematic summaries were developed combining survey and focus group qualitative data.

Quantitative data were analysed using Stata (version 18.0), χ^2^, or Fisher’s exact test to examine associations between participant responses such as demographic characteristics and clinical approach. Where few GPs reported a particular characteristic (for example, non-binary GPs), these were removed from comparative analysis.

Finally, at the interpretation stage, trends in quantitative data were compared with qualitative themes, identifying similarities and differences.

### Reflexivity

The research team were a GP, a midwife, and maternity researchers, all White British and female. Focus group participants were aware of facilitators’ clinical backgrounds. Public contributors with experience of maternity services were included in analytical discussions, in keeping with best practice.^16^

## Results

### Qualitative results

Fourteen GPs participated in four focus groups (Table 1), and 18 women participated in four focus groups (Table 2). GP participants quickly, without prompting from researchers, discussed topics from all areas in the NHSE contract and most NICE recommendations. Discussions reflected their experience, usual practice, or template use; guidance was not cited. Women’s focus groups required prompting and discussion to explore experiences, most accounts being initially very generic with uncertainty about whether the appointment had been about her or only her baby. Six themes around content and timing of the 6–8 week check were identified, which were present in both women’s and GPs’ data.

**Table 1.** Characteristics of GP focus group participants

Characteristics of GPs (*n* = 14)	*n*
Years qualified	
N/A (GPST)	1
≤5	5
6–10	2
11–20	4
≥21	2
GP job role	
Locum	1
Partner	2
Salaried	10
Trainee	1
Is a parent	
Yes	11
No	3
Gender	
Female	13
Male	1
Location	
East Midlands	5
North East	1
London	2
West Midlands	6
Deprivation level of practice^a^	
Affluent	2
Deprived	9
Mixed	2
Ethnic diversity of practice^a^	
Very little diversity, mostly White British	8
Very diverse, multi-ethnic	2
Mixed	3

^a^ *n* = 13, not applicable to locum GP. GPST = general practice specialty trainee

**Table 2.** Characteristics of women focus group participants

Characteristics of women (*n* = 18)	*n*
Age^a^	
21–24	1
25–29	8
30–34	5
35–40	3
Ethnicity	
African	1
Arab	1
Bangladeshi	2
Chinese	1
English, Welsh, Scottish, Northern Irish, or British	4
Indian	4
Other Asian background not specified	1
Other White background not specified	1
Pakistani	3
Number of children	
1	12
2	6
Long-term condition before pregnancy	
Yes	3
No	15
Developed diabetes, high blood pressure, or mental health problem during pregnancy	
Yes	6
No	12
Type of birth	
Caesarean section	11
Instrumental	2
Vaginal	5

^a^ *n* = 17, one participant did not disclose age

#### Physical health and recovery

Physical health and recovery topics, such as vaginal bleeding, pelvic health, and follow-up of pregnancy-related complications, were raised in all focus groups. Women described various experiences of management of their physical health and recovery, with expectations sometimes not met, particularly regarding physical examination:

*’When I was pregnant I had a lot of pelvic floor problems … I asked her* [the GP]*, "I don’t think it was back to normal, I think I’ve got a prolapse"… I wanted her to actually check with my pelvic floor, she was like, "No, just give it a few more weeks and then you can either just call us."… I went in with the expectation of actually checking to see if there was anything wrong.’* (WFG2)

GPs also expressed uncertainty about whether they consistently and sufficiently covered some aspects of physical health and recovery:

*’One of the things that I think it should be about, but I’m not sure it really is at the moment, is about the mum’s health in terms of pelvic floor, pelvic girdle, abdomen, continence. I think a lot of us probably ask about that sort of thing but I think that’s probably something they could do more.*’ (GPFG1)

GPs sometimes placed heavy emphasis on importance of capturing physical health issues during the check because they had sole responsibility:

*’ … to see any ongoing issues from pregnancy, as in hypertension or diabetes, and follow them up that way, because yes you have the midwives and the health visitors, but we tend to be the only medical professionals trying to carry that through, and if we miss that, that would be the last opportunity in a way throughout the postnatal time.’* (GPFG2)

Women and GPs both reported difficulties following up complications of pregnancy and/or birth because of poor communication from the maternity unit where the woman had given birth:

*’… he* [GP] *didn’t even know, it was me that made him aware. Again it’s lack of communication, the lack of transferring data, it’s wrong.’* (WFG1)

’[The birth discharge summary from the hospital] *Doesn’t tend to include pregnancy complications which require ongoing follow-up such as diabetes or hypertension.’* (GPSur222)

#### Mental wellbeing

Mental wellbeing and identifying mental health problems was raised by every GP group and framed as high importance:

*’Maternal mental health is obviously a big one, because I think that’s one of the ones that mums don’t necessarily volunteer themselves.’* (GPFG1)

Many, but not all, women recalled being asked about their mental health. Some were certain they were not asked, some said they’d raised it themselves. Many women in one focus group had telephone consultations and they reported mental health as less prominent. GPs described physically seeing women as forming part of their mental health assessment.

*’I tend to find I focus a lot on mental health aspect, and it’s amazing how people look okay when they’re walking through the room, and then just completely breakdown the minute you ask them how they’re doing.’* (GPFG2)

When women reported mental health problems that required addressing, various solutions were offered, principally signposting to other healthcare professionals such as the health visitor or general mental health services:

*’I am not sure, I can’t remember. But I was low though after I delivered, and they said … they gave me some contacts to get in contact with for my mental health.’* (WFG1)

#### Infant feeding

Infant feeding was raised in three GP focus groups, often in the context of the infant physical examination. There was one suggestion that infant feeding was not integral to the maternal check as it was covered in the baby check, but other GPs described routinely including and responding to feeding-related issues:

*’I have probably over the last few months picked up about six/seven people that I have referred to a lactation consultant for some extra support with breastfeeding.’* (GPFG1)

Women reported being asked about feeding, but also feeling rushed when they fed their baby during the consultation, or not being given support to continue breastfeeding when they wanted to. Sometimes breastfeeding problems were dismissed by the GP:

*’I tried to go in and speak to the doctor about the pain and things like that, and he was like, "Yeah, it* [breastfeeding] *does hurt, it probably does hurt," and then he tried to say, when I said about him not doing it, he went, "Yeah, boys are lazy," and stuff like that. I am just like what are you on about? What? So I just gave up basically.’* (WFG4)

#### Holistic care

GPs spoke positively about the importance of the consultation being holistic and about understanding the woman in the context of her wider family and support network:

*’I think we have had it hammered home a bit in training that it’s also I guess well a good thing, which is you’re getting an opportunity to form a relationship with a new family and hopefully encourage them to seek help appropriately and things.’* (GPFG4)

Women also described general enquiries and discussion from GPs, but sometimes they recalled their consultation as being limited to a general, open question, not extending further:

*’Apart from "how are you generally?" there wasn’t any specific questions to me.’* (WFG2)

#### Health promotion and welfare

Contraception was the predominant feature of this theme, but a range of health promotion topics were raised by GPs and women such as return to exercise and cervical screening. While every GP focus group described the check as a fundamental opportunity to identify safeguarding issues, such as domestic abuse, this was not identified by women.

Discussion of contraception was consistently raised by GPs as important, but several women said this was not covered. Women were uncertain whether it was possible to arrange contraception during the appointment. Some were expecting it to be discussed, but it was not:

*’So my GP didn’t mention contraception, but because the health visitor told me the GP is going to cover the contraception part with you, I’ve actually asked* [the GP].’ (WFG2)

#### Timing of the postnatal check

In the women’s groups, there was no clear consensus regarding the timing of the check. Some who had medical complications, such as infection, would have preferred an earlier review. Many checks happened beyond the 6–8 weeks, although this was viewed positively by some:

*’The fact that it was a lot later I think it wasn’t too much of an issue, and in fact I preferred to be a little bit later. You know initially you have lots of healthcare professionals involved anyway, … it was nice to then have a little touch base again with a healthcare professional at ten to twelve weeks to say "Oh how are things going, is everything okay?"’* (WFG2)

Six to eight weeks was described as too late for issues such as breastfeeding or contraception but one participant’s practice had developed a solution to address this:

*’Too late from a contraceptive point of view, so we’ve now started sending out texts when we get the letter to say that they’ve had their baby, and when we’ve arranged their appointment we also now send out a text to just say that they potentially are fertile from 21 days, so if they want to make an appointment to discuss contraception before their six-week check please do so.’* (GPFG2)

Overall, there were accounts in favour of earlier or later time-points for the check, but 6–8 weeks was described as a good middle-ground, and a suggestion that women would present sooner if needed:

*’In my 20 plus years of experience I would say that most women who have postnatal issues present to me before their 6 weeks check.’* (GPSur378)

### Quantitative survey results

In total, 671 GPs completed the questionnaire (Table 3). The following results are reported with denominators reflecting the number of participants who answered each question. Every geographical region in England was represented. Most responders were partners (*n* = 311/671, 46%) or salaried GPs (*n* = 272/671, 41%). Participants were mostly female (*n* = 548/668, 82%), and parents (*n* = 561/671, 84%). Most (*n =* 608/670, 91%) reported that a universal GP 6–8 week postnatal check was ‘very important’ or ‘absolutely essential’; this was higher for female compared with male responders (*n* = 509/548, 93%, versus *n* = 95/117, 81%*; P*<0.001). Thirty-seven per cent (*n* = 249/671) were unaware of national recommendations for postnatal check content. The majority (*n* = 466/665, 70%) used an electronic template to record the consultation, mostly Ardens.^17^

**Table 3.** Characteristics of GP survey participants

Characteristics of survey participants (*n* = 671)	Participants responding		Total responders, *n*
	***n***	**%**	
**Years qualified**			671
N/A (GPST)	35	5	
≤5	159	24	
6–10	166	25	
11–20	180	27	
≥21	131	20	
**GP job role**			671
Locum	45	7	
Partner	311	46	
Salaried	272	41	
Trainee	35	5	
Other (for example, military GP)	8	1	
**Gender**			668
Female	548	82	
Male	118	18	
Non-binary	1	<1	
Prefer not to say	1	<1	
**Is a parent**			671
Yes	561	84	
No	109	16	
Prefer not to say	1	<1	
**Location**			671
East of England	64	10	
London	75	11	
Midlands	225	34	
North East and Yorkshire	126	19	
North West	50	7	
South East	64	10	
South West	67	10	
**Setting of practice**			669
City	242	36	
Town	207	31	
Rural	91	14	
Mixed	129	19	
**Deprivation level of practice**			669
Affluent	84	13	
Mixed	413	62	
Deprived	172	26	
			
**List size of practice**			657
≤3000	5	1	
3001–10 000	231	35	
10 001–20 000	329	50	
≥20 001	92	14	
**Ethnic diversity of practice**			671
Very little diversity, mostly White British	335	50	
Mixed	207	31	
Very diverse, multi-ethnic	129	19	
**Aware of national guidance on the 6–8 week check**			671
Yes	422	63	
No	249	37	
**Uses a template to document the consultation**			665
Yes	466	70	
No	189	28	
Don’t know	10	2	

Table 4 summarises GPs’ reported delivery of 26 clinical activities taken from NICE recommendations^3^ in postnatal consultations. In the discussion-based activities, contraception, mental health, and recognising physical problems were most commonly ‘always’ undertaken (*n* = 647/671, 96%; *n* = 551/671, 82%; *n* = 455/671, 68%, respectively). Discussions about lifestyle factors (such as diet, exercise, smoking), fatigue, and pelvic floor exercises, were most commonly ‘rarely’ or ‘never’ undertaken (*n* = 150/671, 22%; *n =* 120/671, 18%; *n* = 115/671, 17%, respectively).

Table 5 details associations between GPs’ reported frequency of completion of the 26 clinical activities and GP characteristics where *P*<0.05. Female GPs reported more frequently completing 14/26 clinical activities than male GPs, and GPs who were parents reported more frequently completing 5/26 clinical activities than non-parents. GPs who used a template more frequently completed 17/26, GPs who were aware of national guidance more frequently completed 22/26. GP job role and years of experience were associated with completion of some clinical items, but these didn’t demonstrate a consistent pattern in the way other demographic variables did. For example, GPSTs and GPs with ≤5 years’ experience more frequently discussed safeguarding issues than more experienced GPs, but GPs with the most experience more frequently discussed fatigue. Awareness of national guidance was significantly associated with clinical template use (*P*<0.001); 77% (*n* = 317/412) of those aware of guidance used templates compared with 23% (*n* = 95/412) who did not use templates (Supplementary Data).

**Table 4.** GP reported frequency of completion of 26 clinical items taken from NICE guidance for the routine 6–8 week consultation

	Always		Very often		Sometimes		Rarely		Never	
**Discussion about:**	*n*	**%**	*n*	**%**	*n*	**%**	*n*	**%**	*n*	**%**
Mental health	551	82	75	11	36	5	7	1	2	0.3
Recognising physical problems	455	68	122	18	70	10	20	3	4	1
Pelvic floor exercises	241	36	166	25	149	22	86	13	29	4
Fatigue	157	23	179	27	215	32	100	15	20	3
Diet, exercise, smoking, etc	152	23	179	27	190	28	126	19	24	4
Contraception	647	96	21	3	2	0.3	1	0.2	0	0
Sexual intercourse	324	48	151	23	130	19	58	9	8	1
Safeguarding	252	38	152	23	186	28	69	10	12	2
Birth experience	388	58	147	22	93	14	35	5	8	1
**Specific history taking for:**										
Infection	226	34	140	21	198	30	91	14	16	2
Pain	250	37	167	25	171	25	69	10	14	2
Vaginal loss	476	71	105	16	57	8	27	4	6	1
Bladder function	285	42	134	20	158	24	79	12	15	2
Bowel function	267	40	121	18	179	27	82	12	*22*	*3*
Breast problems	264	39	161	24	161	24	71	11	14	2
Thromboembolism	36	5	80	12	209	31	264	39	82	12
Pre-eclampsia	51	8	67	10	169	25	269	40	115	17
Wound healing	459	68	148	22	42	6	18	3	4	1
Anaemia	103	15	155	23	251	37	129	19	33	5
Psychological wellbeing	585	87	71	11	14	2	1	0.15	0	0
**Clinical examination of:**										
Signs of possible VTE	17	3	14	2	130	19	290	43	220	33
Blood pressure	219	33	122	18	227	34	85	13	18	3
Vaginal problems	50	7	73	11	324	48	169	25	55	8
Caesarean section wound (when applicable)	290	43	204	30	152	23	18	3	7	1
Perineal wound (when applicable)	146	22	179	27	246	37	79	12	21	3
**Review and plan follow-up for:**										
Pregnancy complications, for example, diabetes, hypertension	442	66	163	24	58	9	8	1	0	0

NICE = National Institute for Health and Care Excellence. VTE = venous thromboembolism.

**Table 5.** Associations between 26 clinical items from NICE recommendations and GP characteristics; differences reported where *P*<0.05. In all cases for gender, female GPs reported higher frequency. In all cases for parent status, parent GPs reported higher frequency. In all cases for ‘awareness of guidance’, GPs who were aware of national guidance on the 6–8 week check reported higher frequency. In all cases for ‘use of template’, GPs who used templates to document the consultation reported higher frequency. Specific associations between ‘years of experience’ and ‘role’ varied and are described in the comments column. (Contingency tables in the Supplementary Data.)

	Years of experience	Role	Gender	Parent	Awareness of guidance	Use of template	Comments
**Discussion about:**							
Mental health	-	*-*	-	-	-	-	
Recognising physical problems	-	*-*	-	-	<0.001	0.002	
Pelvic floor exercises	-	*-*	<0.001	0.002	<0.001	<0.001	
Fatigue	0.000	0.039	0.003	<0.001	<0.001	0.046	more frequent in 11–20 and ≥21 years more frequent in partners and locums
Diet, exercise, smoking, etc	-	0.002	-	-	<0.001	<0.001	more frequent in locums and GPSTs
Contraception	-	*-*	0.006	-	-	-	
Sexual intercourse	-	*-*	<0.001	0.004	<0.001	<0.001	
Safeguarding	0.000	-	-	-	<0.001	<0.001	more frequent in GPST and ≤5 years
Birth experience	-	*-*	<0.001	-	0.009	0.042	
**Specific history taking for:**							
Infection	-	*-*	-	-	0.001	0.009	
Pain		*-*	-	-	<0.001	<0.001	
Vaginal loss	-	*-*	<0.001	-	0.003	0.017	
Bladder function	-	*-*	<0.001	-	<0.001	0.003	
Bowel function	-	*-*	0.003	-	<0.001	0.024	
Breast problems	-	*-*	0.001	-	0.001	-	
Thromboembolism	-	*-*	-	-	<0.001	<0.001	
Pre-eclampsia	-	*-*	-	-	<0.001	0.004	
Wound healing	-	*-*	<0.001	-	0.039	-	
Anaemia	0.013	*-*	-	-	<0.001	0.001	more frequent in GPST and ≥21 years
Psychological wellbeing	-	*-*	<0.001	-	0.032	-	
**Clinical examination of:**							
Signs of possible VTE	0.004	*-*	-	-	<0.001	-	more frequent in GPST and ≥21 years
Blood pressure	-	*-*	-	0.024	<0.001	<0.001	
Vaginal problems	-	*-*	<0.001	-	-	-	
Caesarean section wound (when applicable)	-	*-*	-	-	-	-	
Perineal wound (when applicable)	0.049	0.019	<0.001	0.001	0.032	-	more frequent in GPST more frequent in GPSTs and locums
**Review and plan follow-up for:**							
Pregnancy complications, for example, diabetes, hypertension	-	*-*	0.025	-	0.002	0.015	

GPST = general practice specialty trainee. NICE = National Institute for Health and Care Excellence. VTE = venous thromboembolism

Some GPs (*n* = 100/671, 15%) ‘rarely’ or ‘never’ examined a perineal wound when applicable and there was a significant association with gender: (*n =* 43/118) 36% of male versus (*n =* 55/548) 10% of female GPs (*P*<0.001). There was no association between gender and likelihood of examining a caesarean section wound when applicable.

**Figure 1.**
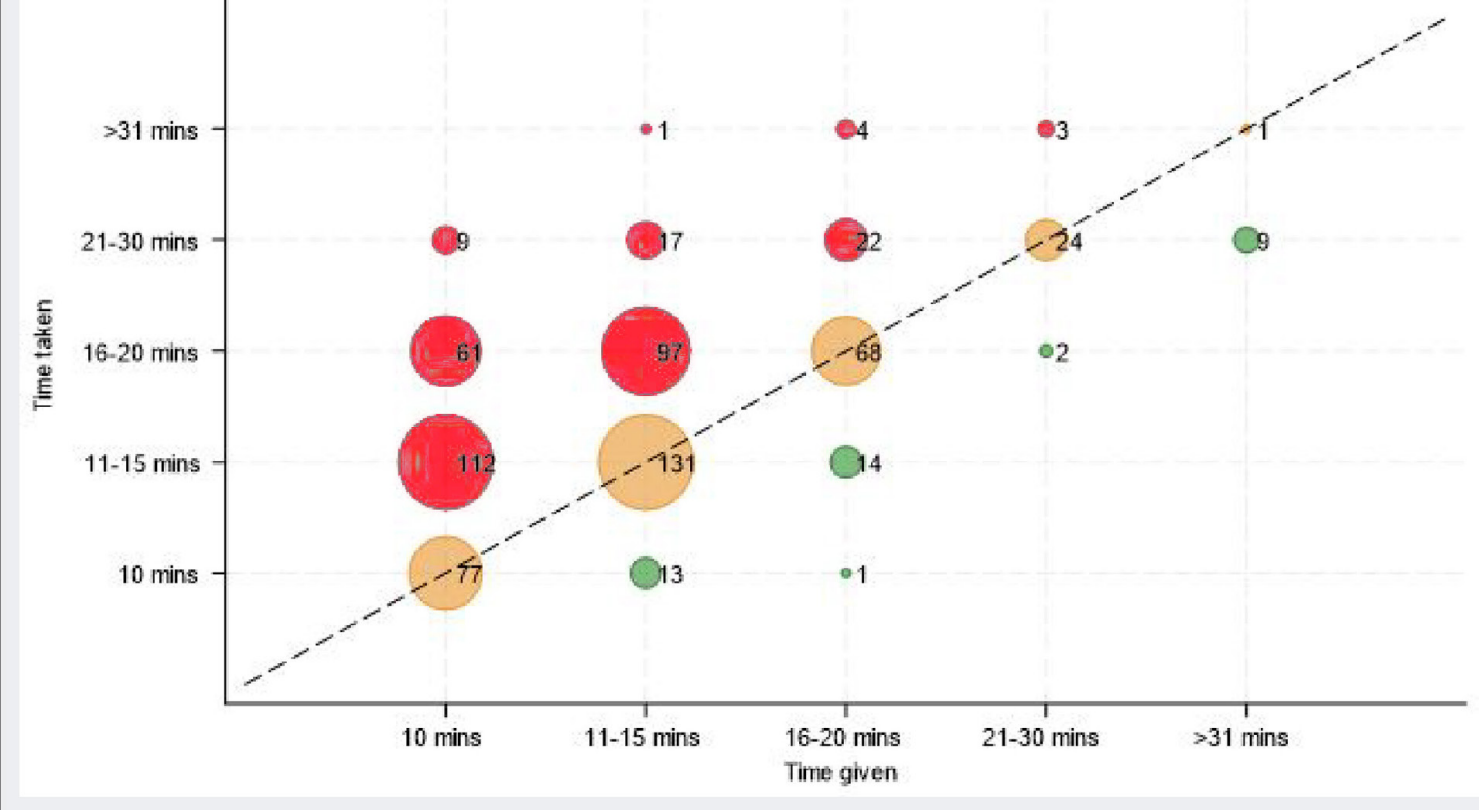
Time taken and given for GP postnatal care appointments. Green points represent where the time taken is less than the time given (below the dotted line); orange points represent when the time taken is the same as the time given (intersecting the line); and red points represent when the time taken is more than the time given for appointments (above the line). Frequency weights are used

Reported time allocated for consultations by GP practices for the maternal consultation (separate to baby checks) varied from none to >31 minutes. Most were allocated 10 minutes (*n* = 263/670, 39%) or 11–15 minutes (*n* = 261/670, 39%) (Supplementary Table 1). Many (*n =* 326/670*,* 49%*)* GPs spent more time than was allocated (Figure 1). Overall, 78% (*n* = 524/670) were allocated ≤15 minutes and 52% (*n* = 351/670) reported completing checks in ≤15 minutes. Female GPs reported requiring longer consultations than male GPs. Template use was associated with consultation length (*P*<0.001); those who used a template took more time compared with those who did not. A choice of GP gender was provided by 48% (*n* = 319/668), a choice of GP was provided by 44% (*n* = 294/670) of participants, and 91% (*n* = 612/669) provided face-to-face consultations, with most others providing a mixture of modalities (*n =* 38/669, 6%).

## Discussion

### Summary

Findings showed alignment between the clinical topics women and GPs perceived as most important in 6–8 week postnatal checks, but not consistent inclusion of these by all GPs. GPs’ emphasis on holistic care contrasted with women’s reports that the GP only asked them how they were generally. Survey data showed that GP awareness of guidance, using a clinical template, being female, and being a parent were associated with more frequently completing more clinical items recommended by NICE. GP participants consistently reported discussing mental health problems with no demographic variation. Optimum timing for the postnatal check was unclear, but for some women (for example, those with medical complications), and some topics (for example, contraception), 6–8 weeks may be too early, or too late.

### Strengths and limitations

In focus groups, views and experiences of women and GPs were explored contemporaneously, allowing interpretation at the same timepoint since COVID-19 restrictions lifted, and since GP contract change. Focus groups varied in size from two to eight participants; potentially, smaller groups may have had reduced opportunity for the development of ideas and viewpoints between participants, but the inclusion of other larger groups and expert facilitation and reflection should mitigate this. This is the first study to explore GP views about their role since the postnatal consultation became mandatory in England. The survey obtained the views and reported clinical practice of many demographically diverse GPs, although 82% were female compared with 57% of the GP workforce in England,^18^ which may reflect greater interest in women’s health and maternity by female GPs. Despite a small incentive and minimising time-commitment, it is likely that GP focus group participants were more interested in postnatal care and offering a higher standard of this care than other GPs. However, some negative responses regarding provision of postnatal care from participants suggests that diverse experiences and perspectives were included. GP data were potentially impacted by social desirability bias, whereby GPs described providing more thorough or compassionate care than in reality.^19^

There was ethnic diversity among women participants, with relatively few White women (*n* = 5). This is relevant given ethnic disparities in risk (significantly higher for Black, Asian, and Mixed ethnicity women) perinatally.^10^ Staff from settings where focus groups were held reported participants were typical of their usual clients; it is therefore likely that women were comfortable in familiar surroundings with peers, and that their accounts were potentially more detailed and reflective, providing important findings for often underserved groups.

### Comparison with existing literature

Findings demonstrated an association between GP gender and approach to care similar to that described in a 1998 Australian study.^12^ This study described routine examination without specific indication, which is no longer recommended in modern practice. In contrast, our findings suggest that examination may currently occur less frequently than indicated, particularly vaginal and perineal examination, especially by male GPs. Similarly, female GPs providing longer consultations is previously described^20^ and not unique to postnatal checks. The higher proportion of female GPs participating in this research may suggest a higher level of engagement in postnatal care as a clinical area. Many male participants did place a high value on, and describe competence in postnatal care, but these may have been a more interested minority.

GPs and women highlighted the same topics as taking priority and these also align with findings from a recent cross-sectional study.^21^ However, there was a mismatch in GP and women’s reports of assessment of physical and mental health. Findings from women were consistent with previous surveys suggesting many are not adequately or specifically asked about mental health, but this was not reported by GPs. Surveys also reported low satisfaction with the time GPs spend on mental and physical health,^5,6^ while GPs in our study reported often spending longer than allocated.

GPs who used a template to record their consultations completed more clinical items but also required more time. Adherence to postnatal guidance was also improved by template use in a US hospital setting.^22^ Use of templates is well-established in UK primary care for long-term condition reviews but concerns have been highlighted about their potential to disrupt communication and prioritise clinician over patient agendas.^23^ Templates developed in collaboration with patients, incorporating an initial open question may reduce this risk.^24^ Time spent on clinical documentation by GPs is reportedly around 14%^25,26^ and increased burden of record-keeping potentially reduces time spent on direct patient care.

Disparities in provision and uptake of routine postnatal care for women and children have been highlighted, and have called into question the clarity of purpose of routine checks for parents.^4,27,28^ This is consistent with the finding in this study that women were unclear about whether their appointment was for them or their baby.

### Implications for research and practice

Most GPs were aware of guidance on the 6–8 week consultation, and used clinical record templates as a rubric. The increased time spent by GPs who used a template, and that they completed a greater number of clinical items, suggests potential benefits to template use, but more time is required to be allocated for appointments. Ultimately, mechanisms to improve safety and achieve greater clinical depth are likely to have a time cost and given widespread preferential use of templates by GPs, they should be kept up to date as guidance changes.

Many GPs needed more time than allocated; it is not surprising that those who spent less time also completed fewer clinical recommendations. Female GPs reported doing more during the consultation and requiring more time. Given the number of clinical tasks within guidance, more than 10 minutes is likely required to address everything sufficiently. The gender differences are relevant from a workforce and leadership perspective; while more GPs are female, more primary care leadership roles are held by men,^29^ who therefore have greater potential to influence factors such as appointment duration. GPs who were allocated more time did spend longer; policy specifying sufficient appointment length and templates to support consultations may improve guidance adherence. Overrunning clinics and desire for longer appointments is well described in UK general practice,^30^ not just postnatal care. The increasing ‘hidden workload’^31^ and professional frustration with contract imposition^32^ potentially create a difficult environment for GPs to fulfil contractual requirements.

Universal provision of a 6–8 week check intends to provide a degree of equity, ensuring no women are missed, but policy should reflect that some groups are at greater risk postnatally.^10^ This study is the first to document the reported clinical practice of GPs in the 6–8 week postnatal maternal consultation and to analyse this with, and in the context of, contemporaneous data from women. Routine postnatal consultations remain important to women, to GPs, and politically.^33^ Clarity about consultation duration and supporting women to know what to expect may improve experiences and outcomes.

## Data

The dataset relied on in this article is available from the corresponding author on reasonable request.
